# Beyond HbA_1_c: insulin resistance as a modifier of early vascular injury in adolescents with type 1 diabetes

**DOI:** 10.3389/fendo.2026.1938241

**Published:** 2026-08-27

**Authors:** Jungin Yoon, Jiyul Kim, Seongjun Park, Woojae Kim

**Affiliations:** 1 Western Reserve Academy, Hudson, OH, United States; 2 Phillips Academy, Andover, MA, United States; 3 Department of Surgery, Beth Israel Deaconess Medical Center, Harvard Medical School, Boston, MA, United States

**Keywords:** arterial stiffness, cardiovascular risk biomarkers, endothelial dysfunction, insulin resistance, type 1 diabetes

## Abstract

Type 1 diabetes (T1D), which commonly presents in childhood or adolescence, is an autoimmune disease in which immune-mediated destruction of pancreatic β-cells leads to absolute or near-absolute insulin deficiency and lifelong dependence on insulin administration. During adolescence, pubertal changes and increased insulin requirements can worsen glycemic instability and raise the risk of vascular complications, such as cardiovascular disease. Although hyperglycemia promotes vascular injury, different early vascular phenotypes in adolescents with similar hemoglobin A_1_c (HbA_1_c) levels suggest that additional mechanisms may influence vascular risk. Insulin resistance (IR) may be an important contributor because insulin sensitivity declines during puberty, and this decline is associated with oxidative stress, altered endothelial signaling, inflammation, and adiposity. However, establishing a causal relationship between IR and early vascular injury in T1D remains challenging. This review examines the link between IR and early vascular injury in adolescents with T1D and emphasizes endothelial dysfunction, arterial stiffness, and biomarkers that may connect metabolic stress to vascular damage. Evidence from metabolic, endothelial, inflammatory, omics-based, and imaging studies supports an association between IR, inflammatory pathways, endothelial injury, and impaired vascular repair. Overall, IR, inflammatory, endothelial, and vascular imaging measures may complement HbA_1_c when studying vascular risk in adolescents with T1D. Whether they provide additional diagnostic or prognostic value beyond HbA_1_c is still unknown.

## 1 Introduction

T1D is primarily an autoimmune disease rather than a lifestyle-driven disorder, although diet, physical activity, adiposity, and other environmental factors may influence metabolic control and vascular risk. T1D typically appears in childhood or young adulthood, and earlier onset is associated with greater long-term risk of severe complications, such as all-cause and cardiovascular mortality (1–5). One reason for this may be the physiological reduction in insulin sensitivity during puberty, which occurs alongside changes in body composition, increased insulin needs, and greater complexity of disease management (6–8). During this transition period, early metabolic stress may contribute to measurable vascular alterations before overt complications appear (2, 9). This may help explain why adolescents with similar HbA_1_c levels can show different cardiovascular risk trajectories.

IR develops when body tissues respond less effectively to insulin therapy. It has increasingly been considered an important modifier of vascular risk in adolescents with T1D (3, 6, 7). However, the available evidence is scattered across inflammation studies, pediatric endocrinology, vascular imaging, and biomarker research. As a result, determining which factors are most biologically relevant, clinically useful, and suitable for integration into a coherent adolescent-specific framework remains challenging. Greater integration of these domains may clarify how IR relates to early vascular dysfunction and may help identify biomarkers for future risk stratification.

This review examines the link between IR and early vascular injury in adolescents with T1D. The main focus is on endothelial dysfunction, arterial stiffness, and biomarkers that may connect metabolic stress to early vascular injury.

## 2 Methods/literature search strategy

This narrative review synthesized pediatric, adolescent, and young adult literature on IR, endothelial dysfunction, vascular imaging, inflammatory biomarkers, and early cardiovascular risk in T1D. We searched PubMed and Google Scholar using combinations of terms “type 1 diabetes,” “adolescents,” “insulin resistance,” “estimated glucose disposal rate,” “endothelial dysfunction,” “flow-mediated dilation,” “pulse wave velocity,” “carotid intima-media thickness,” “biomarkers,” “adipokines,” “microRNA,” and “cardiovascular risk.” Pediatric and adolescent studies were prioritized and adult and mechanistic studies were included when pediatric evidence was limited or when they clarified biological pathways relevant to the review question. The literature search was updated through July 2026 to identify recent studies related to IR, endothelial dysfunction, and vascular complications in T1D. PubMed was used as the primary database, while Google Scholar was used as an additional source for interdisciplinary and recently published literature. Because this was a narrative review, the search was intended to cover a broad range of relevant studies rather than be exhaustive.

Studies were included if they were peer-reviewed and addressed insulin resistance, endothelial dysfunction, vascular injury, biomarkers, or cardiovascular risk in T1D. Priority was given if studies involved pediatric, adolescent, and young adult populations with T1D, especially those that evaluated insulin sensitivity, estimated glucose disposal rate (eGDR), vascular imaging, inflammatory markers, adipokines, endothelial injury and repair, glycemic variability, or cardio-renal risk. Major pediatric cohort studies such as the SEARCH for Diabetes in Youth (SEARCH) study and the Adolescent Type 1 Diabetes Cardio-Renal Intervention Trial (AdDIT) were included because of their relevance to vascular and cardio-renal risk in young people with T1D. Two authors assessed study eligibility together, and any disagreements were resolved through discussion.

Studies were not included if they were not written in English, were not peer reviewed, were limited to case reports, or did not address insulin resistance, vascular dysfunction, biomarkers, or cardiovascular risk in T1D. Although the review focused on T1D, selected studies involving type 2 diabetes (T2D) or obesity were included when they helped clarify mechanisms related to IR, adiposity, endothelial dysfunction, or “double-diabetes” phenotype.

Findings were synthesized descriptively across mechanistic and translational domains such as pubertal insulin resistance, inflammation and oxidative stress, endothelial dysfunction, vascular imaging, biomarker evidence, and risk stratification. Because this was a narrative review rather than a systematic review or meta-analysis, no formal risk-of-bias assessment was performed. The findings should therefore be interpreted as a narrative synthesis rather than a formal assessment of the strength of the evidence.

## 3 Insulin resistance in adolescent patients with T1D

### 3.1 Role of puberty and adiposity

During adolescence, many studies have reported a physiologic decline in insulin sensitivity and have linked it to changes in body composition, pulsatile growth hormone secretion, and increased sex steroid activity (6–8). This shift is common during adolescence, even in youth without obesity, but adolescents with and without T1D respond differently. Healthy teenagers without diabetes partially compensate for their transient decline in insulin sensitivity by increasing endogenous insulin secretion (10). By comparison, adolescents with T1D may be less able to compensate for pubertal IR because they have limited or absent beta-cell reserves and may show substantially lower insulin sensitivity than non-diabetic peers (6, 11).

In parallel, IR may be amplified by the increased prevalence of overweight and obesity in youth with T1D through lipotoxic pathways. In these pathways, excess circulating lipids and lipid intermediates accumulate in non-adipose tissues such as skeletal muscle, liver, myocardium, and vascular tissue (12). Excess free fatty acids and ectopic lipid accumulation can generate lipid intermediates such as ceramides, which activate serine kinase signaling and inhibit insulin receptor substrate-1 (IRS-1)/Akt-mediated glucose uptake (13). Higher insulin dose requirements and frequent treatment of hypoglycemia can contribute to weight gain, which may worsen IR and complicate metabolic management. However, insulin therapy remains essential in T1D (7, 8, 10, 11, 13–15).

### 3.2 Inflammation, oxidative stress, and endothelial IR

In patients with T1D, IR remains an important metabolic abnormality because it can complicate glycemic control, increase daily insulin needs, and is associated with higher cardiovascular and microvascular risk (11). Several studies have identified chronic low-grade inflammation and oxidative stress as potential contributors to IR. Persistent hyperglycemia promotes the formation of advanced glycation end-products (AGEs) through nonenzymatic glycation and oxidation of proteins, lipids, and nucleic acids. These changes can increase reactive oxygen species (ROS) generation and contribute to mitochondrial dysfunction. Together, these processes may impair both insulin signaling and endothelial function (7, 13).

Pubertal and adiposity-related effects may also promote IR in lean youth with T1D. Furthermore, circulating cytokines such as interleukin-6 (IL-6) and tumor necrosis factor-α (TNF-α), which are often reported at higher levels in individuals with T1D, may contribute to impaired insulin signaling, higher insulin dosage requirements, and reduced endothelial repair capacity (7, 16). Recent pediatric studies suggest that elevated high-sensitivity C-reactive protein (hs-CRP), a marker of systemic inflammation produced by the liver, is associated with less favorable metabolic profiles in adolescents with T1D. This supports low-grade inflammation as a clinically detectable correlate of less favorable metabolic status in this population (16, 17).

Systemic IR in adolescents with T1D may be further influenced by endothelial dysfunction. In this setting, impaired vascular responses reduce insulin delivery to peripheral tissues. Reduced nitric oxide (NO)-dependent vasodilation may restrict capillary recruitment and limit insulin transport across the vascular endothelium (18, 19). Adult and mechanistic studies support the concept that impaired microvascular insulin responses reduce skeletal muscle insulin delivery and may contribute to systemic IR, although direct pediatric evidence remains limited (14, 18, 19). This suggests that vascular dysfunction may contribute to whole-body IR by impairing microvascular insulin delivery.

**Figure 1:**
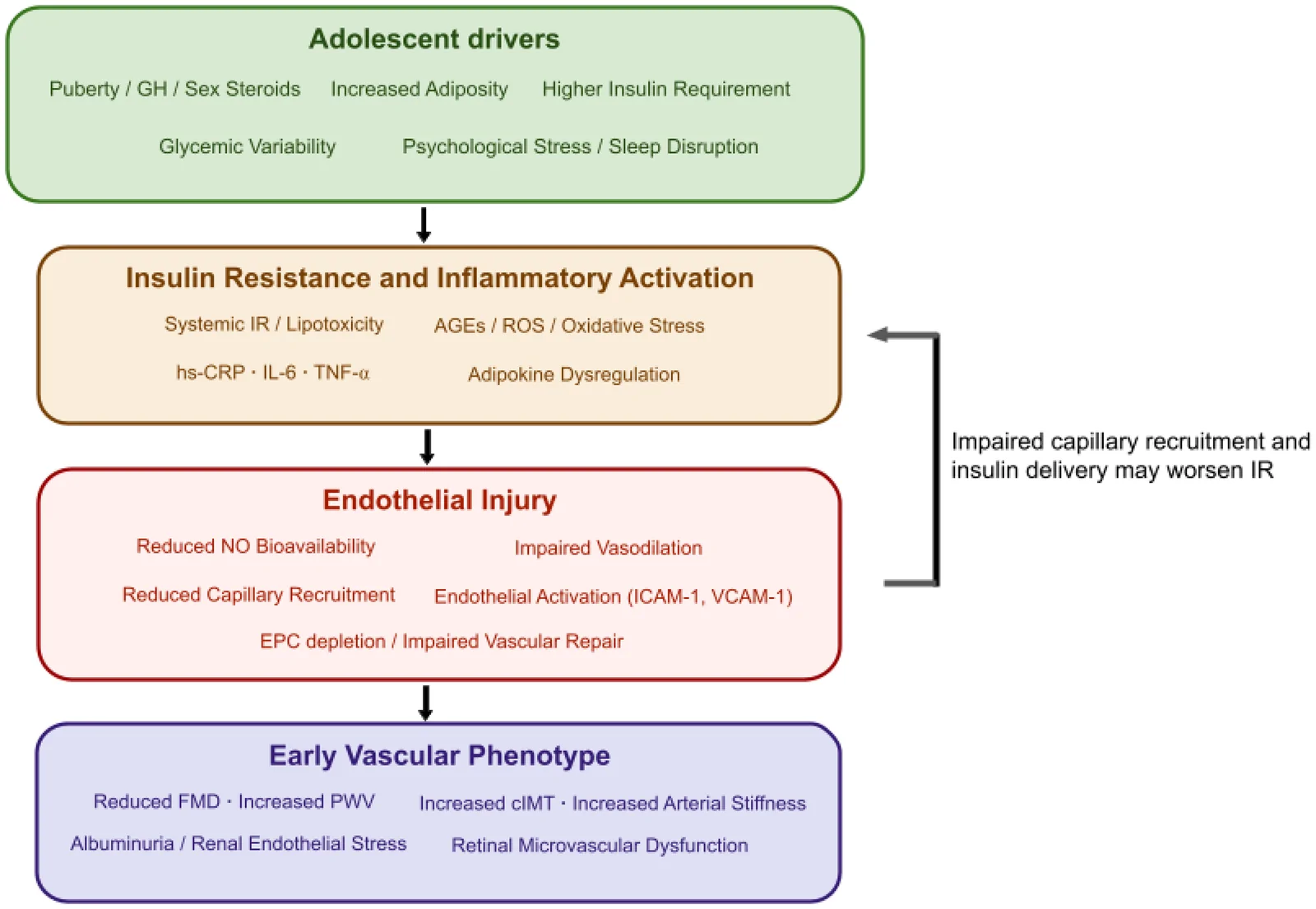
Proposed mechanistic model that links adolescent-specific drivers of insulin resistance to early vascular injury in adolescents with T1D. The downward pathway illustrates the possible contribution of insulin resistance and inflammatory activation to endothelial injury. The return arrow indicates the hypothesized effect of impaired capillary recruitment and insulin delivery on insulin resistance. This bidirectional relationship has not been established prospectively. GH, growth hormone; AGEs, advanced glycation end-products; ROS, reactive oxygen species; hs-CRP, high-sensitivity C-reactive protein; IL-6, interleukin-6; TNF-α, tumor necrosis factor-alpha; NO, nitric oxide; ICAM-1, intercellular adhesion molecule-1; VCAM-1, vascular cell adhesion molecule-1; EPCs, endothelial progenitor cells; FMD, flow-mediated dilation; PWV, pulse wave velocity; cIMT, carotid intima-media thickness.

Therefore, IR in adolescents with T1D is influenced by multiple mechanisms, and understanding how these mechanisms interact may help clarify how vascular risk develops during adolescence. **Figure 1** illustrates the proposed mechanistic model that links these adolescent-specific drivers to early vascular injury and shows the bidirectional relationship between endothelial dysfunction and IR.

### 3.3 Insulin sensitivity assessment and distinction from T2D

Insulin sensitivity is often assessed with the use of the hyperinsulinemic-euglycemic clamp procedure which while reliable is impractical to be used in children or teenagers with T1D due to the complications involved in the procedure and the amount of time that would be needed for it (11). This necessitated the use of alternate methods like eGDR which are based on commonly available clinical data like HbA_1_c levels, anthropometric data and blood pressure of the patient. eGDR uses routinely available clinical variables, including HbA_1_c, anthropometric measures, and hypertension status, to estimate insulin sensitivity. It was originally developed and validated against clamp-derived insulin sensitivity in adults with childhood-onset T1D (20). Although eGDR has subsequently been applied in younger populations, pediatric thresholds and prospective predictive value has yet to be validated (7, 21).

Fasting serum insulin and HOMA-IR are commonly used to assess IR in individuals with preserved endogenous insulin secretion. In established T1D, however, these measures are difficult to interpret because circulating insulin is influenced by exogenous insulin treatment, while endogenous insulin secretion may be minimal or absent. Clamp-derived measures and T1D-specific indices such as eGDR are therefore generally more appropriate (22).

Another possible indirect method to contextualize insulin requirement and glycemic burden in adolescents with T1D is insulin-dose-adjusted HbA_1_c (IDAA1c). Because IDAA1c was developed to evaluate residual beta-cell function and partial remission, it should be interpreted cautiously when used to contextualize IR or glucose management in T1D (23). Assessments of insulin sensitivity in adolescents must account for pubertal stage, body composition, and ethnicity because these factors independently influence surrogate indices such as eGDR and IDAA1c. These influences affect the validity of comparisons across pediatric studies (7, 24).

IR is classically associated with T2D, where genetic susceptibility, excess adiposity, physical inactivity, age, and β-cell dysfunction contribute to disease development. However, IR is also increasingly recognized in T1D. In T2D, IR initially triggers compensatory hyperinsulinemia as beta cells increase insulin output. Over time, progressive beta-cell dysfunction leads to worsening hyperglycemia. In T1D, the IR mechanism follows a different path because it develops on the background of autoimmune loss of insulin production and the body’s reduced responsiveness to administered insulin. This can require higher daily insulin doses to control blood sugar levels. IR in individuals with T1D is associated with genetics, weight gain, chronically elevated blood glucose, puberty, chronic inflammation, increasing adiposity, and higher insulin dose requirements (7, 11).

For example, obese adolescents with T2D may show marked hepatic IR together with reduced levels of adiponectin, an adipocyte-derived hormone involved in metabolic regulation, insulin sensitivity, and inflammation. By comparison, individuals with T1D may show normal or elevated adiponectin concentrations, although interpretation varies by adiposity, disease duration, and renal involvement (16, 25). Elevated adiponectin in T1D should not necessarily be interpreted as protective, as they may reflect compensatory signaling, renal involvement, or altered clearance. However, the rising prevalence of obesity in adolescents with T1D makes this distinction less clear (12). As a result, a “double-diabetes” phenotype has emerged. In this phenotype, a patient with autoimmune T1D develops IR, weight gain, and metabolic characteristics typically associated with T2D (7). This combined phenotype may increase adolescents’ risk of vascular complications, which makes early recognition and targeted intervention important for diabetes management and long-term outcomes.

## 4 Early vascular dysfunction in T1D

In T1D, early signs of vascular dysfunction are often functional in nature. These signs include impaired vasodilation and increased arterial stiffness. These features may appear before overt structural abnormalities or clinically apparent atherosclerosis. In adolescents with T1D, chronic hyperglycemia and inflammatory mediators can reduce NO bioavailability through oxidative stress and inflammation. This contributes to impaired vasodilation that can be assessed through flow-mediated dilation (FMD) (14, 26). Reduced brachial artery FMD and attenuated microvascular reactivity are therefore considered early indicators of vascular dysfunction. In children and adolescents with T1D, meta-analytic studies have shown significantly lower FMD than healthy controls (27) and increased carotid intima-media thickness (cIMT), a marker of early arterial remodeling (28). A recent meta-analysis of 80 case-control studies, including 7,492 individuals with T1D and 5,293 controls, identified a broad range of markers associated with early endothelial dysregulation in T1D. The authors also noted substantial heterogeneity across the included studies (29).

Subclinical vascular dysfunction can be assessed through techniques such as FMD, cIMT, and pulse wave velocity (PWV) (27, 28, 30). Other methods such as high-frequency ultrasound, ultrafast PWV, magnetic resonance imaging (MRI)-based assessment of aortic strain, and retinal vessel analysis can provide complementary assessments of central and microvascular function (31). Retinal microvascular studies in diabetes have shown associations between altered vessel caliber and impaired flicker-light responses. These findings support retinal vascular assessment as a potential measure of microvascular dysfunction (32).

Many studies have shown an association between vascular impairment, IR, and overall metabolic risk. MRI studies have indicated an association between reduced insulin sensitivity and greater aortic stiffness, lower aortic strain, and decreased aortic distensibility (31). Similar patterns have been reported in longitudinal pediatric data from SEARCH and related cohort studies (33, 34). For example, youth with lower insulin sensitivity have been found to display greater progression of arterial stiffness over time (33). Adiposity moderates these associations. Some associations attenuate after adjustment for body mass index (BMI), but adiposity does not eliminate them. This suggests a contribution of IR to vascular function that is at least partially independent of adipose burden (35). Collectively, the data support an association between IR and early vascular dysfunction and indicate that insulin sensitivity is an important correlate of vascular health (36).

Some renal risk markers may become detectable within the early years after T1D diagnosis, although the timing and type of abnormality vary among cohorts (37). In this context, AdDIT and related studies indicated a strong association between higher urinary albumin excretion, even within the normal range, and less favorable cardio-renal profiles. This pattern suggests early renal endothelial stress and higher cardiovascular risk (9, 38). These adolescents showed greater cIMT progression, higher systolic blood pressure, elevated hs-CRP, and greater progression toward albuminuria over time. These findings may reflect early renal microvascular stress (9, 38). Accordingly, adolescents with early renal endothelial stress may display both vascular and inflammatory abnormalities. This is consistent with shared underlying mechanisms that involve chronic IR, endothelial stress, and glycemic variability (39).

Microvascular complications may also occur during adolescence. A recent global meta-analysis of youth with diabetes reported pooled prevalence estimates in T1D of 13.8% for retinopathy, 13.7% for nephropathy, 22.1% for peripheral neuropathy, and 32.0% for autonomic neuropathy (40). The analyses showed substantial heterogeneity, and meta-regression did not identify a significant association between complication prevalence and diabetes duration (40). Nevertheless, broader longitudinal evidence indicates that the risk of microvascular complications increases with longer disease duration and cumulative glycemic exposure (41).

Age and disease duration also play a role. A subset of youth with T1D may show detectable vascular or cardio-renal abnormalities by mid to late adolescence, particularly with greater cumulative risk factor burden. Some studies have reported more abnormal vascular measures in youth with higher HbA_1_c values or hypertension (34), while those with more favorable metabolic profiles displayed normal or near-normal vascular measures.

A key limitation across this literature is the absence of standardized pediatric reference datasets for FMD, PWV, and cIMT that account for age, sex, and pubertal stage. This limits cross-study comparisons (27, 28, 30). FMD is particularly sensitive to protocol variation, and reproducibility has been shown to differ substantially across laboratories (30). Data interpretation in adolescent cohorts is also complicated by the influence of sex and pubertal stage on vascular measures (8, 10).

## 5 Biomarkers of the IR–vascular axis

In adolescents and young adults with T1D, various biomarkers are considered measurable indicators of metabolic stress, vascular damage, and inflammation. These biomarkers may have value to study cardiovascular disease risk and links between IR and vascular outcomes (3, 42). **Table 1** provides a summary of candidate biomarkers and vascular measures.

**Table 1:** Candidate biomarkers and vascular measures associated with insulin resistance and early vascular dysfunction in adolescents with type 1 diabetes.

Domain	Examples	Main interpretation	Pediatric evidence strength	Clinical readiness	Representative evidence
Inflammation/oxidative stress	hs-CRP, IL-6, TNF-α, MDA, 8-isoprostane, oxidized LDL	Low-grade inflammatory activation and oxidative stress; associated with less favorable metabolic profiles and possible early endothelial injury in adolescent T1D	Moderate; mostly cross-sectional evidence from small cohorts	hs-CRP most feasible; others mostly research-use	(7, 16, 17, 43)
Adipokines/metabolic regulators	Adiponectin, leptin, resistin, apelin, chemerin	Reflects adipose dysfunction, compensatory metabolic signaling, and possible “double-diabetes” phenotype; interpretation varies by adiposity and pubertal stage	Mixed to moderate; inconsistent across cohorts	Exploratory; adiponectin/leptin ratio promising but not standardized	(16, 25, 44–46)
Endothelial injury and repair	EPCs, circulating endothelial cells, endothelial microparticles, ICAM-1, VCAM-1, E-selectin	Direct evidence of endothelial damage and impaired repair capacity; EPC depletion correlates with vascular dysfunction measures	Moderate mechanistically; limited clinically	Research-use only; assay standardization needed	(47–52)
Omics/molecular signatures	miR-34a, BCAAs, acylcarnitines, AGEs, skin autofluorescence	Experimental studies link miR-34a to SIRT1-dependent endothelial dysfunction, while metabolomic signatures may reflect altered insulin sensitivity. Pediatric clinical validation is limited.	Exploratory; miR-34a evidence is primarily preclinical, while pediatric metabolomic evidence is limited and largely cross-sectional.	Not ready for clinical screening; skin autofluorescence most accessible	(53–56, 58, 59)
Vascular phenotype/imaging outcomes	FMD, PWV, cIMT, aortic strain/distensibility, retinal vessel analysis	Functional and structural vascular phenotype; most consistent outcome-level evidence associating IR with early vascular dysfunction in adolescent T1D cohorts	Strongest; supported by meta-analytic and longitudinal evidence	Mostly research; highest translational value; FMD and PWV most widely used	(27, 28, 30, 31, 33–35)

Biomarker classes are listed in approximate order from those with the longest clinical history to those with the most exploratory evidence base. Evidence strength reflects the pediatric and young adult literature specifically; adult data may be stronger in some domains. T1D, type 1 diabetes; hs-CRP, high-sensitivity C-reactive protein; IL-6, interleukin-6; TNF-α, tumor necrosis factor-alpha; MDA, malondialdehyde; LDL, low-density lipoprotein; EPCs, endothelial progenitor cells; ICAM-1, intercellular adhesion molecule-1; VCAM-1, vascular cell adhesion molecule-1; miR, microRNA; SIRT1, sirtuin 1; BCAAs, branched-chain amino acids; AGEs, advanced glycation end-products; FMD, flow-mediated dilation; PWV, pulse wave velocity; cIMT, carotid intima-media thickness.

### 5.1 Inflammatory and metabolic biomarkers

Chronic low-grade inflammation, which is reflected by persistent elevations in markers such as cytokines and C-reactive protein, is seen to contribute to vascular injury in diabetic patients. Adolescents and young adults with T1D often face higher inflammatory risk. Prior studies report altered inflammatory markers, such as IL-6 and hs-CRP, in adolescents and young adults with T1D, although findings vary in smaller pediatric studies (7, 16). Adolescents with T1D may also show altered hs-CRP and oxidative-stress measures, but specific markers vary among studies (16, 17, 43). A recent pediatric study reported altered oxidative-stress and antioxidant parameters in adolescents with T1D and supports oxidative stress as a relevant biomarker domain in this population (43). Overall, associations between these factors should be interpreted as suggestive rather than definitive because most pediatric studies related to inflammatory and oxidative biomarkers are relatively small and cross-sectional groups.

One potential early biomarker group to evaluate IR-related metabolic stress in adolescents with T1D is adipokines, which is an insulin-sensitizing and endothelial-protective adipokine. Adiponectin is found at lower levels in T2D and obesity, but adolescents with T1D often show normal or elevated adiponectin concentrations compared with control subjects, although the magnitude varies among different studies (16, 25). These higher adiponectin levels may not entirely counterbalance vascular stress driven by chronic hyperglycemia. Higher adiponectin levels in T1D may also reflect altered metabolic state, disease duration, renal involvement, or early microvascular complications (25).

Leptin concentrations mainly reflect adiposity rather than autoimmune disease status. Leptin levels might be low during diagnosis, rise with insulin therapy and weight restoration, and later correspond more closely with BMI. Higher leptin levels could reflect leptin resistance, which often complements IR in adolescents with obesity (16). Other hormones such as resistin, which is secreted largely by macrophages, are associated with metabolism, obesity, inflammation, and endothelial activation. Elevated resistin concentrations have been observed in individuals with T1D when compared to controls, even under similar BMI conditions (44).

Apelin has been studied as an exploratory adipokine because of its possible role in endothelial NO signaling and metabolic regulation. One pediatric study showed higher plasma apelin concentrations in children with T1D but did not find any association with BMI, lipids, adiponectin, or insulin sensitivity (45). Apelin is therefore discussed as a mechanistic candidate, not as a biomarker for clinical risk stratification.

Other metabolic hormones such as chemerin have been shown to affect glucose uptake, adipogenesis, and inflammatory signaling and may be used as exploratory biomarkers for IR and vascular prediction. Studies in adolescents with T1D have linked higher chemerin levels with cardiometabolic and early vascular risk markers, but these data should be interpreted cautiously because the evidence remains limited (46).

These studies suggest a heterogeneous adipokine profile in adolescents with T1D. Resistin and leptin may contribute to a pro-inflammatory phenotype, while increased production of adiponectin may reflect compensatory or renal factors. There is not enough evidence to include apelin and chemerin in the proposed clinical framework (45, 46). The adiponectin-to-leptin ratio shows promise as a composite research index to identify youth with an emerging “double-diabetes” metabolic profile, but prospective validation in pediatric cohorts is needed before clinical adoption.

### 5.2 Endothelial and cellular biomarkers

Compared to inflammatory and adipose-derived markers, which reflect upstream metabolic stress, endothelial biomarkers offer more direct information about vascular injury and repair capacity. Endothelial progenitor cells (EPCs) are involved in vascular repair, and reduced EPC number or function may reflect impaired endothelial repair (47). Adolescents and young adults with T1D have shown early depletion in EPC number. For example, Dimeglio et al. found individuals aged 18–22 years with T1D to have fewer CD34^+^CD133^+^CD31^+^ progenitor cells and fewer colony-forming EPCs than control subjects (48), along with elevated concentrations of CD34^+^CD45^−^ endothelial cells, which are indicative of active endothelial damage. These studies suggest an association between lower EPC levels and greater endothelial dysfunction, consistent with the relationship between EPC depletion and vascular risk in adults with T1D (47, 49).

Interventions targeting insulin sensitivity may also influence EPC measures. In adults with T1D, 8 weeks of metformin increased circulating EPCs and reduced endothelial injury markers without altering HbA_1_c (50). This adult finding provides mechanistic support, but whether the effect on EPC number specifically is reproducible in adolescents remains untested. Endothelial injury can be detected through endothelial cells and endothelial microparticles (EMPs) as markers. Individuals with T1D have been found to show increased CD34^+^CD45^−^ circulating endothelial cells and EMPs expressing CD31 or CD144, which indicate vascular damage and endothelial dysfunction (48).

Endothelial proteins released from the vascular wall into the circulation may serve as biomarkers of vascular injury, inflammation, and coagulation abnormalities. Pediatric studies have reported higher concentrations of adhesion molecules such as soluble intercellular adhesion molecule-1 (ICAM-1), vascular cell adhesion molecule-1 (VCAM-1), and E-selectin (51, 52). These markers are consistent with endothelial activation and may reflect early microvascular stress. Higher levels of E-selectin and soluble ICAM-1 have also been associated with greater carotid intima-media thickness (cIMT), which connects endothelial activation with subclinical arterial remodeling (51). Other endothelial markers have been evaluated in relation to albuminuria, further emphasizing the connection between endothelial stress, renal microvascular injury, and increased cardiovascular risk (9, 52). These biomarkers may be complemented by circulating immune-cell profiles because youth with type 1 diabetes (T1D) can show a shift toward pro-inflammatory monocyte subsets associated with higher arterial stiffness and increased insulin resistance (35).

Among the biomarkers reviewed, markers of endothelial injury and repair, such as endothelial progenitor cells (EPCs), circulating endothelial cells, endothelial microparticles, and adhesion molecules, are among the most mechanistically relevant to vascular injury. Their clinical use remains limited by assay complexity, limited standardization, and the absence of validated pediatric reference ranges.

### 5.3 Omics and novel molecular biomarkers

Omics approaches in pediatric T1D is still exploratory. miR-34a is mechanistically relevant because studies in diabetic mouse models and endothelial cell systems connect it to endothelial dysfunction through suppression of SIRT1 (53, 54). However, these studies do not establish miR-34a as a biomarker of IR or vascular injury in adolescents with T1D, and including it in this review is based on mechanistic plausibility, not on pediatric clinical validation.

Metabolomic, proteomic, and advanced glycation end-product (AGE)-related measures provide additional but still limited evidence. In adolescents with T1D, altered patterns of branched-chain amino acids (BCAAs), aromatic amino acids, fatty acids, and acylcarnitines may reflect disrupted substrate metabolism and reduced insulin sensitivity (55, 56). Vascular biomarkers studies have suggested a possible role for osteopontin in vascular structure in pediatric T1D and its possible use as an exploratory marker of vascular injury (57). Noninvasive skin autofluorescence studies have shown increased AGE accumulation in adolescents with T1D, with associations to arterial stiffness and long-term glycemic exposure (58, 59). These biomarkers may help clarify molecular mechanisms that connect metabolic stress with endothelial dysfunction, but they are not yet ready for routine clinical use in adolescents with T1D.

## 6 Integrated evidence linking IR to vascular injury in adolescents with T1D

The findings reviewed across metabolic, endothelial, inflammatory, and molecular domains are consistent with a model in which adolescent IR, excess adiposity, chronic hyperglycemia, and oxidative stress may interact to create early vascular dysfunction. In this model, hyperglycemia promotes oxidative stress, which may decrease NO availability, contribute to endothelial injury, and hinder vascular repair. Together, these processes may create measurable arterial dysfunction among adolescents with T1D before the development of overt clinical complications.

Given this, IR may provide information that complements HbA_1_c, although its additional value for vascular-risk prediction has not been established. The presence of divergent vascular phenotypes in adolescents with similar HbA_1_c levels suggests that other factors may contribute to early vascular dysfunction in adolescents with T1D (2, 33, 34).

Evidence from vascular imaging supports this model. Meta-analytic and longitudinal cohort data from SEARCH, AdDIT, and related studies demonstrate associations between lower insulin sensitivity and reduced FMD, increased PWV, greater cIMT progression, decreased aortic distensibility, and clustering of adverse cardiovascular and renal risk markers such as increased urinary albumin excretion (27, 28, 31, 33, 34, 38). Overall, the findings support an association between IR and early arterial dysfunction. However, the evidence is limited by study heterogeneity, relatively small sample sizes, and the absence of a formal risk-of-bias assessment (27, 28, 31, 33, 34, 38). Although adiposity is involved in several links between IR and early vascular dysfunction, it is not the sole unifying factor. Adiposity appears to moderate rather than eliminate these relationships (35).

Circulating and tissue biomarkers have also been studied in relation to this association, but the evidence is less consistent when these markers are grouped together. Chronic low-grade inflammatory markers, such as IL-6 and hs-CRP, support inflammation as a potential mediator of IR leading to endothelial dysfunction, but conflicting results in pediatric studies limit definitive conclusions (7, 16). Adipokine findings are heterogeneous. Elevated adiponectin may reflect compensatory mechanisms, renal involvement, or altered clearance, whereas leptin and resistin may be associated with adiposity and inflammatory activity (16, 44). Regarding biomarkers of vascular injury, EPC depletion, increased circulating endothelial cell numbers, and increased levels of adhesion molecules are markers of active vascular injury and impaired repair capacity. However, the pediatric literature on these biomarkers remains limited (48, 49, 51). Omics-based signatures such as miR-34a and BCAA-related metabolic patterns provide molecular evidence for these associations. However, they have not been well characterized in adequately powered cohorts of adolescents with T1D (53–56). Findings from studies in adults with T1D, individuals with T2D or obesity, and preclinical models may help explain possible mechanisms, but they should not be treated as direct evidence in adolescents with T1D.

One important unresolved issue is bidirectionality. Endothelial dysfunction could develop from IR, but it may also exacerbate IR by negatively affecting microvascular insulin delivery to skeletal muscle and limiting capillary recruitment (14, 18, 19). Therefore, there may be a positive feedback or cyclical relationship between metabolic and vascular dysfunction that helps explain why vascular risk develops in some adolescents with T1D before HbA_1_c levels would fully suggest this risk. However, the existence of this bidirectional relationship has not yet been established in prospective studies. If future research can verify this relationship, it would provide stronger evidence for targeting IR and endothelial function together rather than separately.

It is still unclear whether IR in adolescence comes before early vascular damage, or whether IR and vascular dysfunction share common factors such as chronic hyperglycemia, excess weight, and inflammation that cause them to occur simultaneously or in parallel. Current research shows that there is a link between IR and vascular damage, but evidence for a cause-and-effect relationship would require intervention trials where IR is reduced in adolescents with T1D and vascular outcomes are evaluated over time. Only when there is such evidence, it could be determined if IR is an independent or secondary factor in vascular injury among adolescents with T1D.

## 7 Clinical implications for risk assessment, prevention, and therapy

The assessment of IR and vascular dysfunction in adolescents with T1D has important clinical implications for risk assessment, prevention, and therapy aimed at improving quality of life and long-term outcomes.

### 7.1 Risk stratification of high-risk adolescents with T1D

This framework is conceptual and has not been prospectively validated to be used in clinical risk classification or treatment decisions. **Table 2** summarizes the proposed conceptual risk-stratification framework. Not every adolescent with T1D requires full biomarker assessment, but the evidence shows that those who have increased cardiovascular risk despite acceptable HbA_1_c levels should be identified. The first tier of the model includes parameters that are inexpensive, readily available, and have been shown to have predictive value in T1D. They are HbA_1_c trajectory, BMI z-score, blood pressure (BP), fasting lipid profile, urinary albumin-to-creatinine ratio, and insulin dose per kilogram, and pubertal stage.

**Table 2:** Conceptual risk-stratification framework for early vascular dysfunction in adolescents with type 1 diabetes.

Risk tier	Clinical profile	Biomarker/vascular profile	Interpretation	Appropriate response
Lower-risk phenotype	Lean or normal BMI z-score, active, stable HbA_1_c, normal BP and lipids, low insulin dose requirement, no albuminuria	Low hs-CRP; favorable adipokine profile with higher adiponectin; normal FMD and PWV	No clear clinical evidence of early vascular dysfunction	Continue standard care; reinforce structured exercise, CGM use, and healthy weight trajectory
Emerging metabolic-risk phenotype	Rising BMI z-score, higher insulin dose per kg, pubertal IR, glycemic variability, no overt dyslipidemia or hypertension yet	Higher hs-CRP or IL-6; possible adipokine imbalance with lower adiponectin/leptin ratio; vascular measures may be at lower end of normal	Metabolic vulnerability before overt vascular disease; “double-diabetes” risk if obesity coexists	Intensify lifestyle counseling; review CGM data for variability; consider eGDR trajectory; provide closer follow-up through puberty
Higher vascular-risk phenotype	Hypertension, dyslipidemia, albuminuria, high insulin dose requirement, excess adiposity, longer diabetes duration	Abnormal FMD/PWV/cIMT; inflammatory activation through hs-CRP or IL-6; elevated endothelial injury markers	Early vascular dysfunction likely present; cumulative risk factor burden	Closer follow-up; manage BP, lipids, and albuminuria per standard indications; consider research vascular assessment; intensify all modifiable risk factors
Discordant/research-refinement phenotype	Acceptable HbA_1_c but abnormal vascular or biomarker profile; or omics-level signals without overt clinical risk markers	EPC depletion, elevated endothelial microparticles, miRNA and metabolomic abnormalities; possible subclinical arterial stiffness	Mechanistically important but not clinically standardized; highlights the limitation of HbA_1_c alone as a risk surrogate	Longitudinal research cohort enrollment; avoid overclinicalizing unvalidated biomarkers; continue standard care; document findings for future validation

This framework is proposed for conceptual and research purposes and has not been prospectively validated. Risk tiers are not mutually exclusive and should be interpreted in the context of the full clinical picture. None of the biomarker thresholds listed have established pediatric reference ranges for routine clinical decision-making. Representative evidence supporting the risk domains includes vascular imaging and stiffness studies (27, 30, 33, 34), eGDR and insulin-resistance studies (7, 20, 21), inflammatory and glycemic-variability studies (16, 39), AdDIT/cardio-renal risk studies (9, 38, 65), endothelial injury and repair biomarker studies (47–52), and exploratory omics and metabolomic studies (53–56, 58, 59). Biomarker-guided risk stratification should complement, not replace, standard clinical assessment. HbA_1_c, hemoglobin A_1_c; BP, blood pressure; hs-CRP, high-sensitivity C-reactive protein; IL-6, interleukin-6; miRNA, microRNA; eGDR, estimated glucose disposal rate; FMD, flow-mediated dilation; PWV, pulse wave velocity; cIMT, carotid intima-media thickness; CGM, continuous glucose monitoring; EPCs, endothelialprogenitor cells.

In research settings, adolescents with an adverse first-tier profile, unexplained glycemic variability, or features of the “double-diabetes” phenotype could be considered for second-tier assessment. This second tier may include eGDR, hs-CRP, the adiponectin/leptin ratio, and noninvasive vascular imaging such as FMD or PWV (7, 27, 30). eGDR may be useful as an additional estimate of insulin sensitivity, although pediatric thresholds and prospective predictive value have not been validated. Also, IDAA1c may be a contextually relevant measure of insulin need and glycemic load, but it should be interpreted with caution because its original purpose was to identify partial remission, not IR (23).

Over time, the translational goal is to evaluate whether biomarker and imaging abnormalities observed during adolescent years can predict the development of adult atherosclerosis and cardiovascular events. The adult evidence to date on T1D, especially studies that link low eGDR and inflammatory markers with coronary artery disease and mortality, shows support for developing similar risk models for adolescents with T1D (3, 21, 60). These models may eventually incorporate HbA_1_c trajectory, BMI z-score, BP, lipid profile, hs-CRP, adiponectin/leptin ratio, eGDR, and vascular imaging data into a composite risk score that would need to be validated from adolescence into adulthood (4, 7, 20, 21).

### 7.2 Targeted interventions

The foundation of cardiometabolic risk reduction in adolescents with T1D remains lifestyle optimization through aerobic and resistance exercise, high-quality diet, healthy weight trajectory, and maintaining glycemia within target ranges. Aerobic exercise can increase glucose uptake by skeletal muscle and may improve insulin sensitivity (61, 62). Even when average HbA_1_c values are similar, continuous glucose monitoring (CGM) may help reduce glycemic variability, which has been associated with inflammatory and endothelial markers in adolescent T1D studies (39, 63, 64). Identifying and treating existing elevations in blood pressure, lipids, or albuminuria should occur in accordance with pediatric medical guidelines.

Higher-risk youth include individuals with obesity, high insulin requirements, declining eGDR, or characteristics of the “double-diabetes” phenotype. In these patients, adjunctive metformin therapy may be considered under specialist supervision as a way to decrease overall insulin dosing, limit weight gain, and slightly improve insulin sensitivity (7, 50). The pediatric research base on metformin in T1D is limited, and its effects on HbA_1_c are inconsistent. Additionally, the absence of strong evidence from randomized pediatric studies for direct vascular benefit supports using metformin as a metabolic adjunct rather than as a treatment for vascular disease.

Although statins and angiotensin-converting enzyme (ACE) inhibitors were shown to provide selected secondary benefits in the AdDIT study, the primary albuminuria endpoint was not significantly improved. These findings do not support routine use of ACE inhibitors or statins solely for primary vascular prevention in adolescents with T1D who lack standard clinical indications (65). These agents should generally be used when there is an established indication, such as persistent albuminuria, hypertension, or dyslipidemia.

### 7.3 Psychosocial modifiers

Psychosocial and environmental determinants should not be viewed only as background factors affecting cardiovascular risk in adolescents with T1D. These factors may also act as mediators and participate in the development of vascular dysfunction. For example, chronic psychological stress may elevate cortisol and sympathetic tone and thereby contribute to IR and impaired vascular repair. Similar effects may occur through neuroendocrine dysregulation caused by impaired sleep structure (66). Anxiety, depression, and disordered eating may affect sleep, diabetes management, insulin adherence, physical activity, and glycemic variability in ways that are not fully reflected in HbA_1_c values (66). Substance use, including alcohol, cannabis, and tobacco, may also interfere with treatment adherence and glycemic stability. In one adolescent study, rates of alcohol, tobacco, and cannabis use among youth with T1D were similar to those reported in the general adolescent population, although direct evidence that connect these substances to IR or vascular dysfunction is limited (67). In addition, socioeconomic disadvantage can impair access to appropriate nutrition, physical activity, and advanced diabetes technologies, all of which may affect cardiometabolic outcomes (68–70).

Incorporation of structured assessment of depressive symptoms, sleep disturbances, and food and housing insecurity may improve the care of adolescents with T1D (66, 68, 69). When feasible, behavioral health should become part of the diabetes care team rather than being managed only through a fragmented referral-based approach (66).

Risk stratification strategies should also take equity into account. For example, surrogate measures such as eGDR and hs-CRP are easier to use in settings where resources are limited compared with comprehensive biomarker panels or specialized vascular imaging. Therefore, stratification strategies should be applicable across a wide range of care settings rather than designed only for academic medical centers (68, 70).

Taken together, IR should be considered a factor that may affect susceptibility to vascular damage rather than using glycemic control alone as a marker of cardiovascular risk in adolescents with T1D (3, 42).

## 8 Gaps in current research and future directions

While recognition of the association between insulin resistance and vascular dysfunction in adolescents with type 1 diabetes is increasing, many gaps remain between mechanistic research and clinical practice.

Longitudinal studies that track youth with T1D from diagnosis into adulthood indicate an association between IR and vascular dysfunction. However, because longitudinal evidence remains limited, it is difficult to determine whether IR directly contributes to vascular dysfunction or which adolescents are most likely to develop cardiometabolic complications over time (1, 33). More randomized trials are needed to explore whether treatments directed at IR or inflammation during adolescence improve intermediate vascular outcomes and markers of long-term cardiovascular risk.

Furthermore, researchers have measured IR and vascular function in variable ways because different surrogate indices have been used to assess IR and different protocols have been used to assess vascular function with FMD, PWV, and cIMT techniques (27, 28, 33). Thus, the mismatch between methodology and data interpretation in the literature could be addressed through uniform definitions of IR and vascular dysfunction, as well as reliable measurement procedures that allow for pooled analyses and improved comparability across pediatric studies. These pooled datasets would also need to account for biological factors, such as pubertal stage, that have been shown to significantly affect physiological variation in insulin sensitivity and vascular function (8, 10).

**Figure 2:**
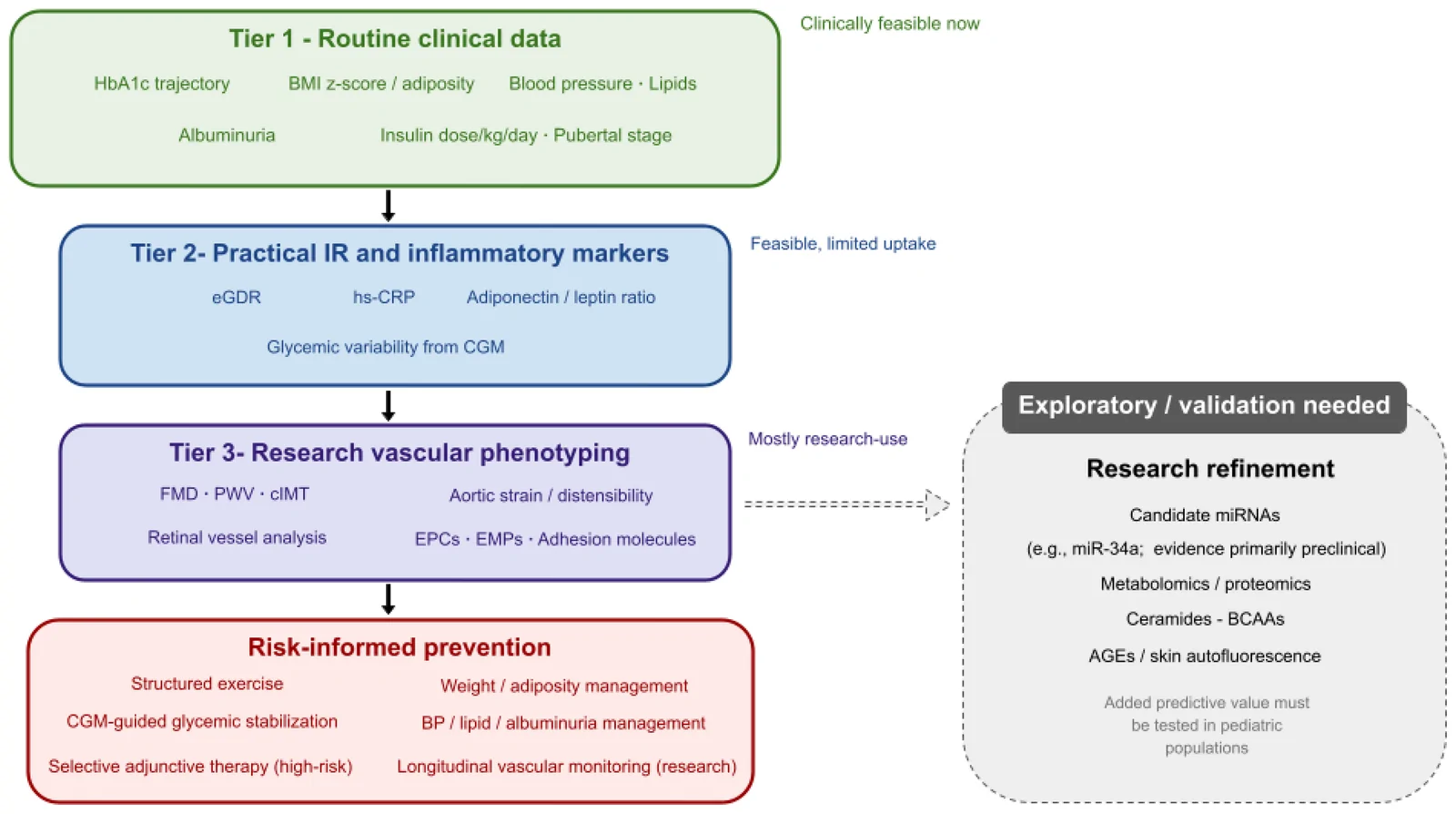
Proposed translational pipeline from routine clinical data through research-grade vascular phenotyping to risk-informed prevention in adolescents with T1D. HbA_1_c, hemoglobin A_1_c; BMI, body mass index; eGDR, estimated glucose disposal rate; hs-CRP, high-sensitivity C-reactive protein; CGM, continuous glucose monitoring; FMD, flow-mediated dilation; PWV, pulse wave velocity; cIMT, carotid intima-media thickness; EPCs, endothelial progenitor cells; EMPs, endothelial microparticles; BP, blood pressure; miRNA, microRNA; BCAAs, branched-chain amino acids; AGEs, advanced glycation end-products.

Biomarker translation remains a major challenge. Commonly studied biomarkers, such as those related to inflammation, endothelial injury, adipose tissue dysfunction, and arterial stiffness, overlap biologically and have often been evaluated through small, cross-sectional studies (47, 48). This challenge could be addressed through multivariate modeling studies that identify smaller sets of biomarkers with incremental predictive value when used together. In practical approaches to early-stage risk categories, composite scores may also be helpful. These scores could combine representative indices from each area, such as hs-CRP for inflammation, eGDR for insulin resistance, and PWV for arterial stiffness (21, 34). **Figure 2** illustrates the proposed translational pipeline from routine clinical data through research-grade vascular phenotyping to risk-informed prevention.

For example, IL-6 assays and endothelial imaging remain only partially accessible at the community level because of cost, logistics, and uncertainty regarding clinical utility. Implementation studies should also evaluate whether identifying adolescents with increased insulin resistance or inflammatory burden supports the initiation of early structured intervention strategies.

Social and structural factors should also be considered when implementing these recommendations. These factors include safe areas for physical activity, access to healthy food, reliable health care, and advanced diabetes technologies (68–70). Equity-focused approaches can reduce disparities, particularly in low-resource settings where simplified surrogate measures may be more reasonable and feasible to track than expensive technologies such as comprehensive biomarker panels for insulin resistance or vascular stress. When technologies are readily available, future research efforts should explore whether biomarker combinations improve the information gained from standard clinical variables such as glucose levels, blood pressure, HbA_1_c, BMI, lipids, and albuminuria (7, 9, 20).

Collaboration among lifestyle medicine, endocrinology, cardiology, vascular biology, and data science may provide new insights from mechanistic studies that evaluate relationships between metabolic, inflammatory, and vascular processes. Larger collaborative consortia, standardized protocols, and longitudinal designs may help researchers study the interaction of metabolic indices, inflammatory markers, and vascular imaging data in adolescents with T1D and develop validated vascular health profiles (33, 34). Together, these efforts may help identify youth at risk for T1D-related cardiovascular disease earlier and support preventive strategies aimed at reducing long-term cardiovascular risk (1, 3, 42).

## 9 Conclusions

This review presents evidence for an association between increased IR during adolescence in T1D and early vascular dysfunction, particularly impaired endothelial function and increased arterial stiffness. Results from vascular imaging, circulating endothelial markers, and molecular studies are consistent with a relationship between IR and early vascular injury. This relationship may help explain why HbA_1_c does not reflect every aspect of early vascular biology in adolescents with T1D. IR-related, inflammatory, endothelial, and molecular measures may provide complementary information, but their additional diagnostic or prognostic value beyond HbA_1_c has not been established. Emerging biomarkers should therefore be exploratory until they are validated in pediatric populations. Future studies should test whether integrating measures of IR, inflammation, vascular function, and selected cellular or omics markers improves risk prediction beyond routine clinical measures. This requires standardized pediatric assessment protocols, larger cohorts, and validated biomarker panels. Therefore, the proposed framework should be regarded as a research model, not a validated clinical risk score.
